# Case-Control Study of Use of Personal Protective Measures and Risk for SARS-CoV 2 Infection, Thailand

**DOI:** 10.3201/eid2611.203003

**Published:** 2020-11

**Authors:** Pawinee Doung-ngern, Rapeepong Suphanchaimat, Apinya Panjangampatthana, Chawisar Janekrongtham, Duangrat Ruampoom, Nawaporn Daochaeng, Napatchakorn Eungkanit, Nichakul Pisitpayat, Nuengruethai Srisong, Oiythip Yasopa, Patchanee Plernprom, Pitiphon Promduangsi, Panita Kumphon, Paphanij Suangtho, Peeriya Watakulsin, Sarinya Chaiya, Somkid Kripattanapong, Thanawadee Chantian, Emily Bloss, Chawetsan Namwat, Direk Limmathurotsakul

**Affiliations:** Ministry of Public Health, Nonthaburi, Thailand (P. Doung-ngern, R. Suphanchaimat, A. Panjangampatthana, C. Janekrongtham, D. Ruampoom, N. Daochaeng, N. Eungkanit, N. Pisitpayat, N. Srisong, O. Yasopa, P. Plernprom, P. Promduangsi, P. Kumphon, P. Suangtho, P. Watakulsin, S. Chaiya, S. Kripattanapong, T. Chantian, C. Namwat);; Thailand Ministry of Public Health–US Centers for Disease Control and Prevention Collaboration, Nonthaburi (E. Bloss);; Mahidol University, Bangkok, Thailand (D. Limmathurotsakul);; University of Oxford, Oxford, United Kingdom (D. Limmathurotsakul)

**Keywords:** respiratory infections, severe acute respiratory syndrome coronavirus 2, SARS-CoV-2, SARS, COVID-19, zoonoses, viruses, coronavirus, coronavirus disease, masks, handwashing, social distancing, contact tracing, personal protective equipment, PPE

## Abstract

We evaluated effectiveness of personal protective measures against severe acute respiratory disease coronavirus 2 (SARS-CoV-2) infection. Our case-control study included 211 cases of coronavirus disease (COVID-19) and 839 controls in Thailand. Cases were defined as asymptomatic contacts of COVID-19 patients who later tested positive for SARS-CoV-2; controls were asymptomatic contacts who never tested positive. Wearing masks all the time during contact was independently associated with lower risk for SARS-CoV-2 infection compared with not wearing masks; wearing a mask sometimes during contact did not lower infection risk. We found the type of mask worn was not independently associated with infection and that contacts who always wore masks were more likely to practice social distancing. Maintaining >1 m distance from a person with COVID-19, having close contact for <15 minutes, and frequent handwashing were independently associated with lower risk for infection. Our findings support consistent wearing of masks, handwashing, and social distancing to protect against COVID-19.

Evaluation of the effectiveness of mask-wearing to protect healthy persons in the general public from infection with severe acute respiratory syndrome coronavirus 2 (SARS-CoV-2), the causative agent of coronavirus disease (COVID-19), is urgently needed (*1*,*2*). On February 27, 2020, during the early stages of the COVID-19 outbreak, the World Health Organization (WHO) announced that wearing a mask of any type was not recommended for asymptomatic persons (*3*). The rationale at that time was to avoid unnecessary cost, procurement burden, and a false sense of security (*3*). Several systematic reviews found no conclusive evidence to support widespread use of masks in public settings to protect against respiratory infectious diseases, such as influenza and severe acute respiratory syndrome (SARS) (*4*–*6*). However, China, South Korea, Japan, Thailand, and other countries in Asia have recommended the use of face masks among the general public since early in the COVID-19 pandemic (*7*). Evidence suggests that persons with COVID-19 can have a presymptomatic period, during which they can be contagious and transmit SARS-CoV-2 to others before symptoms develop (*8*). These findings led to a change in recommendations from the US Centers for Disease Control and Prevention on April 4, 2020, that advised all persons wear a cloth face covering when in public (*9*). On April 6 and June 5, 2020, WHO updated its advice on the use of masks for the general public and encouraged countries that issue the recommendations to conduct research on this topic (*8*).

Thailand has been implementing multiple measures against transmission of SARS-CoV-2 since the beginning of the outbreak (*10*,*11*). The country established thermal screening at airports on January 3, 2020, and detected an early case of COVID-19 outside China in a traveler from Wuhan, China, arriving at Bangkok Suvarnabhumi airport on January 8, 2020 (*10*). Thailand uses Surveillance and Rapid Response Teams (SRRTs), together with village health volunteers, to conduct contact tracing, educate the public about COVID-19, and monitor close contacts of persons with COVID-19 in quarantine (*11*). SRRTs are epidemiologic teams trained to conduct surveillance, investigations, and initial control of communicable diseases, such as SARS and influenza (*12*,*13*). More than 1,000 district-, provincial-, and regional-level SRRTs are working on COVID-19 contact tracing in Thailand.

By February 2020, public pressure to wear masks in Thailand was high. However, medical masks became difficult for the public to procure, and the government categorized medical masks as price-controlled goods. When the Ministry of Public Health (MoPH) designated COVID-19 a dangerous communicable disease, according to the Communicable Disease Act of 2015, government officials were empowered to quarantine case-contacts and close venues (*14*,*15*). On March 3, MoPH recommended public use of cloth face masks (*16*). On March 18, schools, universities, bars, nightclubs, and entertainment venues were closed (*17*). On March 26, when the country was reporting »100–150 new COVID-19 cases per day, the government declared a national state of emergency, prohibited public gatherings, and enforced wearing of face masks by all persons on public transport (*18*). On April 21, MoPH announced 19 new PCR-confirmed COVID-19 cases, bringing the total number of cases to 2,811 (*18*). Given the lack of evidence, we sought to evaluate the effectiveness of mask-wearing, handwashing, social distancing, and other personal protective measures against SARS-CoV-2 infection in public in Thailand.

## Methods

### Study Design

We conducted a retrospective case-control study by drawing persons with COVID-19 cases and noninfected controls from a cohort of contact tracing records of the central SRRT team at the Department of Disease Control (DDC), MoPH, Thailand. We included contact investigations of 3 large COVID-19 clusters in nightclubs, boxing stadiums, and a state enterprise office in Thailand.

Contacts were defined by DDC as persons who had activities with or were in the same location as a person with confirmed COVID-19 (*19*,*20*). The main aim of contact tracing was to identify and evaluate contacts, perform reverse transcription PCR (RT-PCR) diagnostic tests, and quarantine high-risk contacts, as defined by the MoPH (Appendix). RT-PCR was performed at laboratories certified for COVID-19 testing by the National Institute of Health of Thailand (*19*,*20*). Data on risk factors associated with SARS-CoV-2 infection, such as type of contact and use of mask, were recorded during contact investigations, but data sometimes were incomplete.

The central SRRT performed contact investigations for clusters of >5 PCR-confirmed COVID-19 cases from the same location within a 1-week period (*19*,*20*). We used these data to identify contacts who were asymptomatic during March 1–31, 2020. We used all available contact tracing records of the central SRRT in the study.

We telephoned contacts during April 30–May 27, 2020, and asked details about their contact with a COVID-19 index patient, such as dates, locations, duration, and distance of contact. We asked whether contacts wore a mask during the contact with the index patient, the type of mask, and the frequency of wearing a mask, which we defined as compliance with mask-wearing. We asked whether and how frequently contacts washed their hands while with the index patient. We asked whether contacts performed social distancing and whether they had physical contact with the COVID-19 index patient. If they did not know, or could not remember, contact with the index patient, we asked whether they had contact with other persons at the location. We asked whether the contact shared a cup or a cigarette with other persons in the place they had contact or had highest risk for contact with the index patient and whether the COVID-19 index patient, if known to the respondent, had worn a mask (Appendix, Additional Methods). We also asked, and verified by using DDC records, whether and when the contacts had symptoms and when COVID-19 was diagnosed.

For our study, we defined cases as asymptomatic contacts who later tested positive for SARS-CoV-2, on the basis of RT-PCR results available by April 21, 2020. We defined controls as asymptomatic contacts who did not have positive test results for SARS-CoV-2 by April 21, including those who tested negative and those who were not tested. We defined asymptomatic contacts as persons who had contact with or were in the same location as a symptomatic COVID-19 patient and had no symptoms of COVID-19 on the first day of contact. We defined index patients as persons identified from contact tracing data as the potential source of SARS-CoV-2 infection; cases (as defined above) also could be index patients. We defined primary index patients as persons whose probable sources of infection were before the study period, March 1–31; for whom we were not able to identify the source of infection; or whose probable sources of infection were outside the contact tracing data included in the study. We defined high-risk exposure as that which occurred when persons lived in the same household as a COVID-19 patient; had a direct physical contact with a COVID-19 patient; were <1 m from a COVID-19 patient for >15 minutes; or were in the same closed environment, such as a room, nightclub, stadium, or vehicle, <1 m from a COVID-19 patient for >15 minutes.

We used 21 days after March 31 as a cutoff date based on evidence that most persons with COVID-19 likely would develop symptoms within 14 days (*21*) and that it could take <7 additional days for symptomatic persons under contact investigation to go to a healthcare facility and be tested for COVID-19. Our study follows the STROBE guidelines (*22*).

### Statistical Analysis

To include only initially asymptomatic contacts in the study, we excluded persons who reported having symptoms of COVID-19 at the time of initial contact with an index patient. Because our study focused on the risk for infection in the community, we excluded contacts whose contact locations were healthcare facilities. We also excluded primary index patients if they were the first to have symptoms at the contact investigation location, had symptoms since the first day of visiting the location, or were the origin of infection based on the contact investigation.

We estimated secondary attack rates by using the percentage of new cases among asymptomatic contacts with high-risk exposure to enable comparison with other studies. We estimated odds ratios (ORs) and 95% CIs for associations between developing COVID-19 and factors evaluated. We used logistic regression with random effects for location and for index patients nested in the same location. If an asymptomatic contact had contact with >1 symptomatic COVID-19 index patient, the interviewer identified the index patient as the symptomatic COVID-19 patient with the closest contact. The percentage of missing values for the variable indicating whether the index patients wore a mask was 27%; thus, we did not include this variable in our analyses. For other variables, we assumed that missing values were missing at random and used imputation by chained equations (*23*,*24*). We created 10 imputed datasets and the imputation model included the case-control indicator and variables used in the multivariable models, including sex, age group, contact place, shortest distance of contact, duration of contact at <1 m, sharing dishes or cups, sharing cigarettes, handwashing, mask-wearing, and compliance with mask-wearing. We developed the final multilevel mixed-effect logistic regression models on the basis of previous knowledge and a purposeful selection method (*25*; Appendix, Additional Analyses). Because of collinearity between mask use and mask type, we conducted a separate analysis including mask type in the multilevel mixed-effects logistic regression model for SARS-CoV-2 infection. We also tested a predefined interaction between mask type and compliance with mask-wearing (Appendix, Additional Analyses).

To clarify patterns of behavior and factors related to compliance with mask-wearing, we used multinomial logistic regression models and the imputed dataset to estimated OR and 95% CI for associations between 3 mask-wearing compliance categories, never, sometimes, or all the time; and for other practices, including handwashing and social distancing during the contact period. We used logistic regression to estimate p values for pairwise comparisons.

To estimate the proportional reduction in cases that would occur if exposure to risk factors was reduced, we estimated the population attributable fraction by using the imputed dataset and a direct method based on logistic regression, as described previously (*26*,*27*; Appendix, Additional Analyses). We performed all analyses by using Stata version 14.2 (StataCorp, https://www.stata.com) and R version 4.0.0 (R Foundation for Statistical Computing, https://www.r-project.org).

## Results

### Characteristics of the Cohort Data

**Figure 1:**
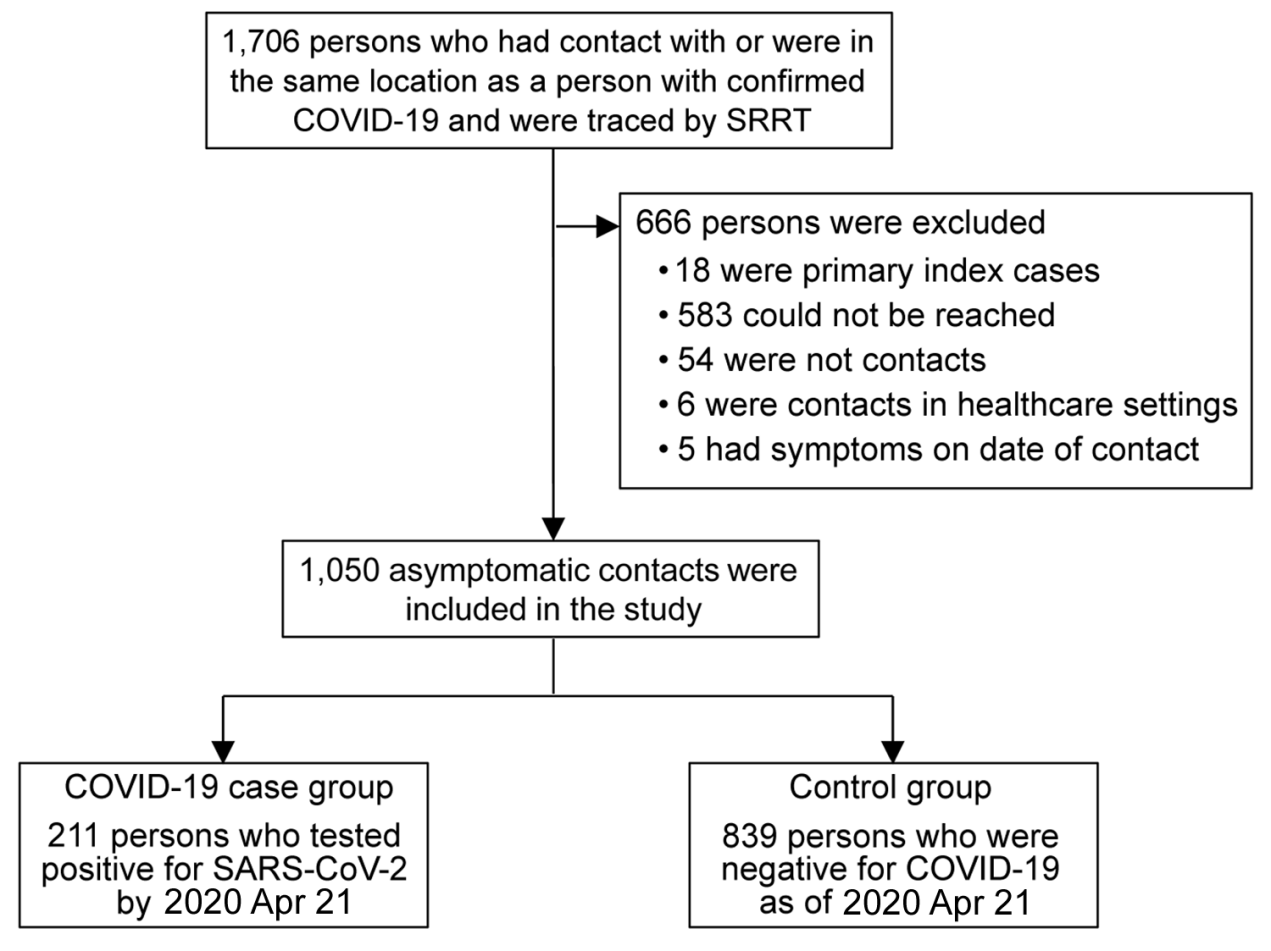
Flow diagram in case-control study of severe acute respiratory syndrome coronavirus 2 infections and contacts, Thailand, March–April 2020. COVID-19, coronavirus disease; SARS-CoV-2, severe acute respiratory syndrome coronavirus 2; SRRT, Surveillance and Rapid Response Team of Ministry of Public Health.

The contact tracing records of the central SRRT included 1,716 persons who had contact with or were in the same location as a person with diagnosed COVID-19 in an investigation of 3 large clusters (Figure 1). Overall, 18 primary index patients were identified: 11 from the nightclub cluster, 5 from the boxing stadiums cluster, and 2 from the state enterprise office cluster. Timelines of primary index patients from the 3 clusters varied (Appendix Figures 1–3); we excluded the 18 primary index patients from our analyses.

### Characteristics of Cases and Controls

After interviewing each contact by phone and applying the exclusion criteria (Figure 1), our analysis included 1,050 asymptomatic persons who had contact with or were in the same location as a symptomatic COVID-19 index patient during March 1–31, 2020. The median age of persons included was 38 years (IQR 28–51 years); 55% were male, and 45% were female (Table 1). Most (61%; n = 645) asymptomatic contacts included in the study were associated with the boxing stadiums cluster, 36% (n = 374) were related to the nightclub cluster, and 3% (n = 31) were related to the state enterprise office cluster. Overall, 890 (84.8%) contacts were considered to have high-risk exposure.

**Table 1:** Factors associated with coronavirus disease among persons followed through contract tracing, Thailand, March–April 2020*

Factors	COVID-19 cases, no. (%), N = 211†	Controls, no. (%), N = 839†	Crude odds ratio (95% CI)‡	p value	Adjusted odds ratio (95% CI)‡	p value
Sex	n = 211	n = 838				
F	65 (30.8)	404 (48.2)	Referent	0.52	Referent	0.37
M	146 (69.2)	434 (51.8)	0.83 (0.47–1.46)		0.76 (0.41–1.41)	
Age group, y	n =211	n = 829				
<15	6 (2.8)	49 (5.9)	0.65 (0.17–2.48)	0.29	0.57 (0.15–2.21)	0.21
>15–40	94 (44.5)	435 (52.5)	Referent		Referent	
>40–65	98 (46.4)	302 (36.4)	1.65 (0.91–2.97)		1.77 (0.94–3.32)	
>65	13 (6.2)	43 (5.0)	1.29 (0.33–5.07)		0.97 (0.22–4.24)	
Contact place§	n = 211	n = 839				
Nightclub	35 (16.6)	193 (23.0)	NA	NA	NA	NA
Boxing stadium	125 (59.2)	19 (2.3)				
Workplace	11 (5.2)	286 (34.0)				
Household	38 (18.0)	192 (22.9)				
Others	2 (0.9)	149 (17.8)				
Shortest distance of contact	n = 197	n = 809				
Physical contact	132 (67.0)	292 (36.1)	Referent	0.001	Referent	0.02
<1 m without physical contact	61 (30.9)	335 (41.4)	0.76 (0.43–1.35)		1.09 (0.58–2.07)	
>1 m	4 (2.0)	182 (22.5)	0.08 (0.02–0.31)		0.15 (0.04–0.63)	
Duration of contact within 1 m	n = 199	n = 801				
>60 min	180 (90.4)	487 (60.8)	Referent	0.003	Referent	0.09
>15–60 min	14 (7.0)	162 (20.2)	0.53 (0.24–1.17)		0.67 (0.29–1.55)	
<15 min	5 (2.5)	152 (19.0)	0.13 (0.04–0.46)		0.24 (0.07–0.90)	
Sharing dishes or cups¶	n = 210	n = 837				
N	125 (59.5)	576 (68.8)	Referent	0.001	Referent	0.39
Y	85 (40.5)	261 (31.2)	2.71 (1.48–4.94)		1.33 (0.70–2.54)	
Sharing cigarettes#	n = 209	n = 836				
N	196 (93.8)	824 (98.6)	Referent	0.001	Referent	0.03
Y	13 (6.2)	12 (1.4)	6.12 (2.12–17.72)		3.47 (1.09–11.02)	
Handwashing**	n = 210	n = 826				
None	44 (20.9)	121 (14.6)	Referent	<0.001	Referent	0.045
Sometimes	114 (54.3)	333 (40.3)	0.41 (0.18–0.91)		0.34 (0.14–0.81)	
Often	52 (24.8)	372 (45.0)	0.19 (0.08–0.46)		0.33 (0.13–0.87)	
Type of mask††	n = 211	n = 834				
None	102 (48.3)	500 (60.0)	Referent	0.003	–	–
Nonmedical masks only	25 (11.8)	77 (9.2)	0.78 (0.32–1.90)			
Nonmedical and medical	12 (5.7)	48 (5.8)	0.46 (0.13–1.64)			
Medical mask only	72 (34.1)	209 (25.0)	0.25 (0.12–0.53)			
Compliance with mask-wearing††	n = 210	n = 823				
Not wearing a mask	102 (48.6)	500 (60.7)	Referent	<0.001	Referent	0.006
Wearing a mask sometimes	79 (37.6)	125 (15.2)	0.75 (0.37–1.52)		0.87 (0.41–1.84)	
Always wearing a mask	29 (13.8)	198 (24.1)	0.16 (0.07–0.36)		0.23 (0.09–0.60)	
*NA, not applicable; COVID-19, coronavirus disease. †Data not available for all cases and controls for all factors. ‡Crude and adjusted odds ratios were estimated using logistic regression with random effects for location and for index patient nested within the same location. §The state enterprise office was included as a workplace. Others included restaurants, markets, malls, religious places, and households of index patients or other persons by persons not living in that household. Location was included in the model as a random effect variable. ¶Sharing multiserving dishes and using communal serving utensils to portion food individually was not categorized as sharing dishes. #Included sharing electronic cigarettes and any vaping devices. **Included washing with soap and water, and by using alcohol-based solutions. ††Wearing masks incorrectly, such as not covering both nose and mouth, was considered the same as not wearing a mask for analyses. Crude odds ratios of wearing mask and of each factor evaluated were estimated using logistic regression with random effects for location and for index patient nested within the same location to take into account clustering; therefore, the crude odds ratios are not equal to dividing of the odds in the case group by the odds in the control group.						

Among 1,050 asymptomatic contacts included in our analysis, 211 (20.1%) tested positive for SARS-CoV-2 by April 21, 2020, and were classified as cases; 839 (79.9%) never tested positive and were controls. Of the 211 cases, 195 (92.4%) had high-risk exposures and 150 (71.1%) had symptoms before COVID-19 diagnosis by RT-PCR (Appendix). The last date that a COVID-19 case was detected was April 9, 2020. Among the 839 controls, 695 (82.8%) had high-risk exposures and 719 (85.7%) were tested by PCR at least once.

**Figure 2:**
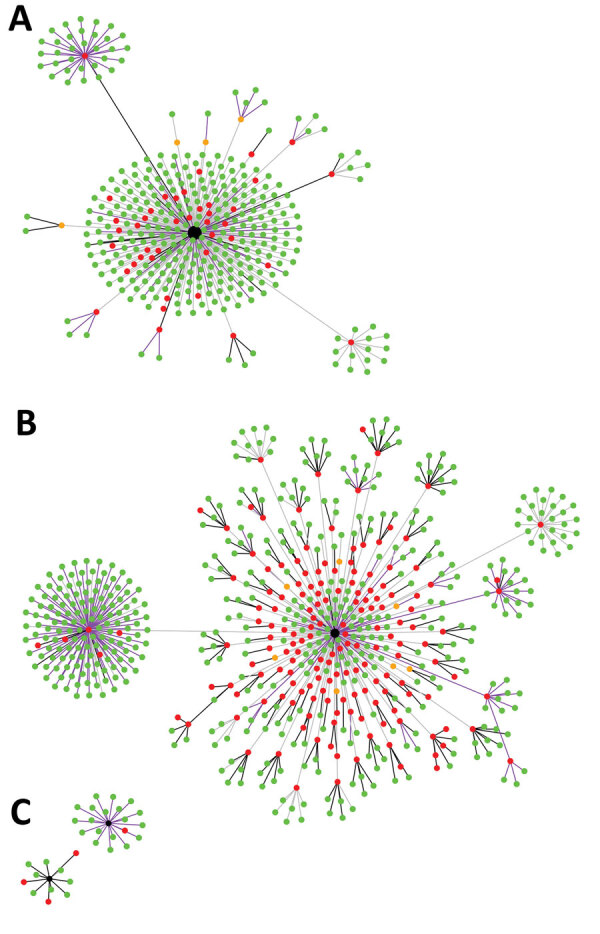
Development and transmission of severe acute respiratory syndrome coronavirus 2 among asymptomatic contacts, Thailand, March–April 2020. Clusters indicate coronavirus disease (COVID-19) contacts from nightclubs (A); boxing stadiums (B), and the state enterprise office (C). Black nodes represent primary index patients, red dots cases (contacts of primary index patients who had COVID-19), green dots noninfected controls, and orange dots patients with confirmed COVID-19 who could not be contacted by the study team. Black lines represent household contacts, purple lines contacts at workplaces, and gray lines contacts at other locations.

Among asymptomatic contacts included in the study, 228 had contact with a COVID-19 index patient at nightclubs, 144 at boxing stadiums, and 20 at the state enterprise office (Figure 2). The others had contacts with a COVID-19 index patient at workplaces (n = 277), households (n = 230), and other places (n = 151). Among 890 asymptomatic contacts with high-risk exposures included in the study, the secondary attack rate from boxing stadiums was 86% (111/129), the secondary attack rate for nightclubs was 18.2% (34/187), the household secondary attack rate was 16.5% (38/230), the workplace secondary attack rate was 4.9% (10/205), and the secondary attack rate at other places was 1.4% (2/139).

### Bivariate Analyses

Our analysis showed risk for SARS-CoV-2 infection was negatively associated with personal protective measures (Table 1). Crude odds ratio (OR) for infection was 0.08 (95% CI 0.02–0.31) for those maintaining a distance of >1 m from a COVID-19 patient; 0.13 (95% CI 0.04–0.46) for those whose duration of contact was ≤15 minutes; 0.41 (95% CI 0.18–0.91) for those who performed handwashing sometimes; 0.19 (95% CI 0.08–0.46) for those who washed hands often; and 0.16 (95% CI 0.07–0.36) for those wearing a mask all the time during contact with a COVID-19 patient. We noted a higher risk for SARS-CoV-2 infection among persons sharing dishes or cups (OR 2.71; 95% CI 1.48–4.94) and sharing cigarettes (OR 6.12; 95% CI 2.12–17.72) with other persons in general, not necessarily including a COVID-19 patient. In the bivariate model, type of mask was associated with risk for infection (p = 0.003).

### Multivariable Analyses

We found a negative association between risk for SARS-CoV-2 infection and maintaining a distance of >1 m from a COVID-19 patient (adjusted odds ratio [aOR] 0.15; 95% CI 0.04–0.63); duration of contact <15 minutes (aOR 0.24; 95% CI 0.07–0.90); handwashing often (aOR 0.33; 95% CI 0.13–0.87); and wearing a mask all the time during contact with a COVID-19 patient (aOR 0.23; 95% CI 0.09–0.60) (Table 1). Wearing masks sometimes during contact with a COVID-19 patient was not statistically significantly associated with lower risk for infection (aOR 0.87; 95% CI 0.41–1.84). Sharing cigarettes with other persons was associated with higher risk for infection (aOR 3.47; 95% CI 1.09–11.02).

Compliance with mask-wearing during contact with a COVID-19 patient was strongly associated with lower risk for infection in the multivariable model. Because of collinearity with mask-wearing compliance, we did not include mask type in the final model. We included mask type in a separate multivariable model and found type of mask was not independently associated with infection (p = 0.54) (Appendix Table 1). We found no evidence of effect modification between mask type and mask-wearing compliance.

### Association Between Mask-Wearing Compliance and Other Social Distancing Practices

Because mask-wearing throughout the contact period was negatively associated with COVID-19, we further explored characteristics of participants to ascertain whether wearing a mask produced a potential false sense of security. We found that during the contact period, 25% of persons who wore masks all the time reported maintaining >1 m distance from contacts compared with 18% of persons who did not wear a mask (pairwise p = 0.03). In addition, persons who wore masks all the time were more likely to report duration of contact <15 minutes (26% vs. 13% for those who did not wear a mask; pairwise p<0.001) and washing hands often (79% vs. 26% for those who did not wear a mask; pairwise p<0.001) (Table 2). We found that 43% of persons who wore masks sometimes were likely to wash their hands often compared with those who did not wear masks (26%; pairwise p<0.001), but they were more likely to have physical contact (50% vs. 42%; pairwise p = 0.03) and report duration of contact >60 minutes (75% vs. 67%; pairwise p = 0.04) compared with those who did not wear masks.

**Table 2:** Factors associated with compliance of mask wearing among coronavirus disease cases and controls, Thailand, March–April 2020*

Factors	Not wearing masks, no. (%), n = 602	Sometimes wearing masks, no. (%), n = 204	Always wearing masks, no. (%), n = 227	p value†
Sex	n = 601	n = 204	n = 227	
F	277 (46.1)	75 (36.8)	112 (49.3)	0.03
M	324 (53.9)	129 (63.2)	115 (50.7)	
Age group, y	n = 594	n = 204	n = 225	
<15	45 (7.6)	5/204 (2%)	3/225 (1%)	<0.001
>15–40	269 (45.3)	117/204 (57%)	132/225 (59%)	
>40–65	236 (39.7)	76/204 (37%)	84/225 (37%)	
>65	44 (7.4)	6/204 (3%)	6/225 (3%)	
Contact places‡	n = 602	n = 204	n = 227	
Nightclub	84 (14.0)	51 (25.0)	91 (40.1)	<0.001
Boxing stadium	48 (8.0)	66 (32.4)	29 (12.8)	
Workplace	178 (29.6)	46 (22.5)	64 (28.2)	
Household	167 (27.7)	27 (13.2)	33 (14.5)	
Others	125 (20.7)	14 (6.9)	10 (4.4)	
Shortest distance of contact	n = 588	n = 191	n = 212	
Physical contact	246 (41.8)	96 (50.3)	76 (35.8)	0.005
<1 m without physical contact	238 (40.5)	70 (36.6)	83 (39.1)	
>1 m	104 (17.7)	25 (13.1)	53 (25.0)	
Duration of contact within 1 m	n = 590	n = 190	n = 205	
>60 min	396 (67.1)	143 (75.3)	121 (59.0)	<0.001
>15–60 min	120 (20.3)	23 (12.1)	30 (14.6)	
<15 min	74 (12.5)	24 (12.6)	54 (26.3)	
Sharing dishes or cups§	n = 601	n = 203	n = 226	
N	361 (60.1)	130 (64.0)	200 (88.5)	<0.001
Y	240 (39.9)	73 (36.0)	26 (11.5)	
Sharing cigarettes¶	n = 600	n = 202	n = 226	
N	586 (97.7)	194 (96.0)	223 (98.7)	0.29
Y	14 (2.3)	8 (4.0)	3 (1.3)	
Handwashing#	n = 594	n = 203	n = 224	
None	142 (23.9)	16 (7.9)	6 (2.7)	<0.001
Sometimes	298 (50.2)	99 (48.8)	42 (18.8)	
Often	154 (25.9)	88 (43.3)	176 (78.6)	
*Data not available for all cases and controls for all factors. Wearing masks incorrectly, such as not covering both nose and mouth, was considered the same as not wearing a mask for analyses. †p values were estimated by using univariable multinomial logistic regression models. Missing values were imputed using the imputation model. ‡The state enterprise office was included as a workplace. Others included restaurants, markets, malls, religious places, and households of index patients or other persons but not living together (e.g., persons not living in that household). §Sharing multiserving dishes and using communal serving utensils to portion food individually was not categorized as sharing dishes. ¶Included sharing electronic cigarettes and any vaping devices. #Included washing with soap and water, and by using alcohol-based solutions.				

## Discussion

Our findings provide evidence that mask-wearing, handwashing, and social distancing are independently associated with lower risk for SARS-CoV-2 infection in the general public in community settings in Thailand. We observed that wearing masks throughout the period of exposure to someone infected with SARS-CoV-2 was associated with lower risk for infection, but wearing masks only sometimes during the period was not. This evidence supports recommendations to wear masks consistently and correctly at all times in public (*2*,*7**–**9*).

The effectiveness of mask-wearing we observed is consistent with previous studies, including a randomized-controlled trial showing that consistent face mask use reduced risk for influenza-like illness (*28*), 2 case-control studies that found that mask-wearing was associated with lower risk for SARS infection (*29*,*30*), and a retrospective cohort study that found that mask-wearing by index patients or family members at home was associated with lower risk for SARS-CoV-2 infection (*31*). Previous studies found use of surgical masks or similar 12–16-layer cotton reusable masks demonstrated protection against coronavirus infection in the community (*32*), but we did not observe a difference between wearing nonmedical and medical masks in the general population. Our results suggest that wearing nonmedical masks in public can potentially reduce transmission of SARS-CoV-2. Another study found perception of risk of developing COVID-19 can increase a person’s likelihood of wearing a medical mask in nonmedical settings (T.D. Huynh, unpub. data, https://www.medrxiv.org/content/10.1101/2020.03.26.20044388v1). However, given supply shortages, medical masks should be reserved for use by healthcare workers.

We found a negative association between risk for SARS-CoV-2 infection and social distancing, consistent with previous studies that found that >1 m physical distancing was associated with a large protective effect and distances of >2 m could be more effective (*32*). Our findings on effectiveness of hand hygiene also were consistent with reports in previous studies (*33*).

In this study, secondary attack rates at different venues varied widely. The household secondary attack rate in our study (16.5%) is comparable with ranges reported previously (11%–23%) (*34*,*35*), and relatively high compared with workplaces (4.9%) and other settings (1.4%). Although quarantine measures can be challenging and sometimes impractical, household members should immediately separate a person who develops symptoms of COVID-19; the sick person should stay in a specific room; use a separate bathroom, if possible; and not share dishes, cups, and other utensils (*36*). All household members should wear masks, frequently wash their hands, and perform social distancing to the extent possible (*37*).

The high number of COVID-19 cases associated with nightclub exposures in Bangkok is comparable to a COVID-19 outbreak associated with the Itaewon nightclub cluster in Seoul, South Korea, during May 2020 (*38*), in which persons visited several nightclubs in the same area during a short period of time. The secondary attack rate in boxing stadiums was high (86%) but similar to a cluster of COVID-19 cases associated with a football match in Italy during February 2020 (*39*). The secondary attack rate of COVID-19 at a choir practice in the United States was reported to be 53.3%–86.7% (*40*). Secondary attack rates in public gathering places with high densities of persons shouting and cheering, such as football and boxing stadiums, are still uncertain but appear to be high.

Clear and consistent public messaging from policy makers likely can prevent a false sense of security and promote compliance with social distancing in Thailand. We found that those who wore masks throughout the time they were exposed to a COVID-19 patient also were more likely to wash their hands and perform social distancing. Traditional and social media outlets can support public health responses by working with governments to provide consistent, simple, and clear messages (*41*). In Thailand, daily briefings from the Centre for COVID-19 Situation Administration provided clear, consistent messages on social distancing, how to put on a mask, and how to wash hands, which might have improved public confidence with the recommendations. Consistent public messages on how to wear masks correctly also are needed, particularly for those who wear masks sometimes or incorrectly, such as not covering both nose and mouth. We found that persons who only intermittently wore masks during exposure also did not practice social distancing adequately.

Our study has several limitations. First, because our findings were based on contacts related to 3 major COVID-19 clusters in Thailand during March 2020, they might not be generalizable to all settings. Second, estimated ORs were conditioned on reported contact with index patients; our study did not evaluate or consider the probability of having contact with other infected persons in the community, which could have occurred. Third, because only 89% of controls were tested, those not tested could have been infected; therefore, cases might have been missed in persons with mild or no symptoms or who did not report symptoms or seek care or testing. Nonetheless, we believe that misclassification likely was minimal because those who were not tested with RT-PCR were low-risk contacts; the small number likely would not change our main findings and recommendations on personal protective measures. Fourth, identifying every potential contact can be nearly impossible because some persons might have had contact with >1 COVID-19 patient. Hence, our estimated secondary attack rates among contacts with high-risk exposure could be overestimated or underestimated. Fifth, population attributable fraction is based on several assumptions, including causality, and should be interpreted with caution (*42*,*43*). Finally, findings were subject to common biases of retrospective case-control studies, including memory bias, observer bias, and information bias (*44*). To reduce potential biases, we used structured interviews in which each participant was asked the same set of defined questions.

As many countries begin to relax social distancing measures, our findings provide evidence supporting consistent mask-wearing, handwashing, and adhering to social distancing recommendations to reduce SARS-CoV-2 transmission in public gatherings. Wearing nonmedical masks in public could help slow the spread of COVID-19. Complying with all measures could be highly effective; however, in places with a high population density, additional measures might be required.

Clear and consistent public messaging on personal protective recommendations is essential, particularly for targeting those who wear masks intermittently or incorrectly. Our data showed that no single protective measure was associated with complete protection from COVID-19. All measures, including mask-wearing, handwashing, and social distancing, can increase protection against COVID-19 in public settings.

Appendix Additional methods, analyses, and discussion on a case-control study of use of personal protective measures and risk for severe acute respiratory syndrome coronavirus 2 infection, Thailand.
