# Supplementary material for: Case-Control Study of Use of Personal Protective Measures and Risk for SARS-CoV 2 Infection, Thailand
Source: Emerg Infect Dis. 2020 Nov;26(11):2607–16. doi: 10.3201/eid2611.203003 (PMC7588529; doi:10.3201/eid2611.203003)
Supplement: Appendix — Additional methods, analyses, and discussion on a case-control study of use of personal protective measures and risk for severe acute respiratory syndrome coronavirus 2 infection, Thailand. [file 20-3003-Techapp-s1.pdf]

Article DOI: https://doi.org/10.3201/eid2611.203003

Case-Control Study of Use of Personal Protective Measures and Risk for SARS-CoV-2 Infection, Thailand

Appendix

Additional Methods

Study Design

Contacts were defined by the Department of Disease Control (DDC), Ministry of Public Health (MoPH), Thailand as persons who had activities with or were in the same location as a COVID-19 patient (*1*,*2*). All high-risk contacts with any symptoms were tested with a reverse transcription PCR (RT-PCR) assay and quarantined in a hospital or a quarantine site. High-risk contacts defined by the MoPH included family members or persons who lived in the same household as a COVID-19 patient; persons ≤1 m distance of a COVID-19 patient for >15 minutes; persons exposed to coughs, sneezes, or secretions of a COVID-19 patient and were not wearing protective gear, such as a mask; and persons in the same closed environment, such as a room, nightclub, stadium, or vehicle, ≤1 m from a COVID-19 patient for >15 minutes, and they were not wearing protective gear, such as a mask (*1*,*2*). All high-risk contacts without any symptoms were self-quarantined at home. Before March 23, 2020, all high-risk contacts without any symptoms were tested by using RT-PCR assays on day 5 after the last date of exposure to a COVID-19 patient (*1*,*2*). As of March 23, 2020, all household contacts were tested by using RT-PCR assays regardless of their symptoms (*1*,*2*). Other high-risk contacts were tested only if COVID-19 symptoms developed. All low-risk contacts were recommended to perform self-monitoring for 14 days and visit healthcare facilities immediately for RT-PCR assays if COVID-19 symptoms developed (*1*,*2*). All RT-PCR assays targeting open read frame 1b (ORF1b) and N gene regions were performed at laboratories certified for COVID-19 testing by the National Institute of Health of Thailand (*3*) based on the protocols recommended by the World Health Organization (WHO; *4*). RT-PCR assays required a detection limit of <25 copies/reaction and no

cross-reactivity with a panel of other respiratory viruses including SARS-CoV, MERS-CoV, and hCoV (NL63, OC43, 229E, and HKU1) (*3*). When an RT-PCR assay result was negative, the result was reported immediately. When a RT-PCR assay result was positive for either or both ORF1b and N gene regions, a confirmation test was conducted by using either a PCR assay targeting different regions or targeting ORF1b and N gene regions with different targets, or nucleotide sequencing of ORF1b or N gene regions.

We telephoned contacts during April 30–May 27, 2020 and asked details about their contact with a COVID-19 index patient, such as date, location, duration, and distance of contact. We asked whether contacts wore a mask during the contact period with the following possible responses: never; yes, non-medical mask; yes, medical mask; yes, alternately nonmedical and medical masks; unknown or cannot remember. If contacts reported wearing a mask, we asked the frequency of mask-wearing during the contact period with the following possible responses: sometimes; all the time; unknown or cannot remember. We asked if and how frequently they washed their hands during the contact period with the following possible responses: no washing with soap or alcohol-based solutions; yes, sometimes; yes, all the time after any contact (defined below as ‘often’); unknown or cannot remember. We asked if they performed social distancing, including type of contact with the COVID-19 index patient or other persons at place of contact, if unable to remember who the patient was with the following possible responses: had physical contact; shortest distance $\leq$1 m and no physical contact; shortest distance >1 m and no physical contact; unknown or cannot remember. We asked about the total duration of contact with the following possible responses: >1 hour; at least 15 minutes but not >1 hour; <15 minutes; unknown or cannot remember. We asked whether contacts shared a cup or a cigarette with other persons in the place they had contact or had highest risk for contact with the index patient with the following possible responses: no, yes, unknown or cannot remember. We asked whether the COVID-19 index patient, if known to the respondent, had worn a mask with the following possible responses: never; yes, nonmedical mask; yes, medical mask; yes, alternately nonmedical and medical masks; unknown or cannot remember. We asked if the COVID-19 index patient wore a mask and the frequency of mask-wearing with the following possible responses: sometimes; all the time; unknown or cannot remember. We also asked, and verified using DDC records, whether and when the contacts had symptoms or COVID-19 was diagnosed. The reporting of this study follows the STROBE guidelines (*5*).

Statistical Analysis

We developed the final multilevel mixed-effect logistic regression models on the basis of previous knowledge and a purposeful selection method (*6*). In short, we performed the following: 1) fit a multilevel mixed-effect univariable model with each covariate; 2) selected candidate variables with the α level of <0.25; 3) evaluated variables that were not statistically significant in the multivariate model at an α level of 0.10; 4) fit a reduced model and evaluated confounding by change in log odds ratios of any remaining variables compared with the full model; 5) repeated steps 3 and 4 until the model contained statistically significant covariates or confounders; and 6) added back in the model, 1 at a time, any variable not originally selected, kept any that were statistically significant, and reduced the model following steps 3 and 4. We kept sex, age group, and sharing dishes or cups in the mixed-effect multivariable model on the basis of previous knowledge that sex, age group, and sharing dishes or cups were associated with COVID-19 development.

We estimated the direct population attributable fraction (PAF) by using the imputed dataset and the direct method, as previously described (*7,8*). Direct PAF can be obtained by calculating PAFs directly from subjects' data by using logistic regression (*7,8*). First, we modified our final logistic regression model by considering each risk factor dichotomously. Then, irrespective of exposure to each risk factor for each subject, that factor was removed from the population by calculating probability based on all observations as unexposed. The predicted probability of developing COVID-19 for each asymptomatic contact, with the assumption that no exposure to a certain risk factor occurred, was defined by:

$$P_{ki} = \frac{1}{1 + \exp\left[-\left(\beta_0 + \sum_{j \neq i} \beta_j x_j\right)\right]}$$

P_{ki} is representative of predicted probability of COVID-19 in an asymptomatic contact, k, assuming no exposure to a specific risk factor (x_i); β_j indicates the regression coefficient of risk factor (x_j), except risk factor number i (x_i). Subsequently, the sum of all predicted probabilities for all subjects in the study would be equal to the adjusted estimate of total cases, which is anticipated in the absence of that specific risk factor (x_i).

Then, PAF was estimated by subtraction of the total number of predicted cases from total number of observed cases, divided by the total number of observed cases:

$$\text{PAF} = \frac{\text{Total number of observed cases} - \text{total number of predicted cases}}{\text{total number of observed cases}}$$

Additional Results

Characteristics of the Cohort Data

For the nightclub cluster, we identified 11 primary index patients who started having symptoms during March 4–8 and had a COVID-19 diagnosis and began isolation during March 3–10 (Appendix Figure 1). Primary index patients visited multiple nightclubs included in the analysis during the study period, and 35/228 (15.4%) asymptomatic contacts at nightclubs had PCR-confirmed SARS-CoV-2 infections after the contact (Figure 2, panel A).

For the boxing stadium cluster, we identified 5 primary index patients who started having symptoms during March 6–12 and had diagnosed COVID-19 and began isolation during March 11–21 (Appendix Figure 2). Primary index patients visited multiple boxing stadiums included in the analysis during the study period, and 125/144 (86.8%) asymptomatic contacts at the boxing stadiums had RT-PCR–confirmed SARS-CoV-2 infections after the contact (Figure 2, panel B).

Among the 2 primary index patients for the office cluster (Appendix Figure 3), 1 had symptoms that began on March 15, 2020 (primary index patient C1) and was considered the source of infection for 1 new case in the office during the study period. The other primary index patient (primary index patient C2) was a household member of a staffer at the office and was considered the source of infection for the staffer via household contact.

Characteristics of Cases and Controls

Because all household contacts were tested with RT-PCR assays, we further explored characteristics of household contacts. Among 38 household contacts who later had diagnosed COVID-19, 20 (52.6%) reported having symptoms before diagnosis by RT-PCR. Among 192 household contacts who did not have diagnosed COVID-19, 9 (4.6%) reported having symptoms ($p<0.001$). The median time from exposure to a COVID-19 index patient and testing by RT-PCR did not differ between household contacts who later had COVID-19 (9 days [IQR 6–14 days]) and household contacts who did not have COVID-19 (9 days [IQR 6–12 days]; $p = 0.65$).

Multivariable Analyses

Wearing masks all the time during contact was independently associated with lower risk for SARS-CoV-2 infection compared with not wearing masks (aOR 0.23; 95% CI 0.09–0.60) (Appendix Table 1), but wearing masks sometimes during contact was not (aOR 0.87; 95%CI 0.41–1.84). We further explored whether the risk for infection was different between those who wore masks all the time and those who wore masks sometimes. We found a negative association between risk for SARS-CoV-2 infection and wearing masks all the time compared with wearing masks sometimes (aOR 0.27; 95% CI 0.11–0.70; p = 0.007).

PAF

We estimated that the proportional reduction in cases that might occur if everyone wore a mask all the time during contact with COVID-19 patients was 0.28; that is, the PAF of not wearing masks all the time (Appendix Table 2). Among modifiable risk factors evaluated, PAF of shortest distance of contact <1 m was highest (0.40). Based on our data, if everyone wore a mask all the time; washed hands often; did not share a dish, cup, or cigarette; had shortest distance of contact >1 m; and had duration of contact <15 min, COVID-19 cases might have been reduced by 84%.

Additional Discussion

We also quantified the effectiveness of different measures that could be implemented to prevent transmission in nightclubs, stadiums, workplaces, and other public gathering places. We estimated that adopting all recommendations (mask-wearing all the time; handwashing often; not sharing dishes, cups, or cigarettes; maintaining a distance of >1 m and, if this distance cannot be maintained, limiting contact duration to <15 minutes) might prevent ≥84% of COVID-19 infections in the study settings during the study period. However, PAF is based on several assumptions, including causality, and should be interpreted with caution (*9*,*10*). Nonetheless, public gathering places could consider multiple measures to protect against COVID-19 and new pandemic diseases in the future.

References

1. Thailand Ministry of Public Health, Department of Disease Control. Coronavirus disease 2019: COVID-19, updated on 3 March 2020 [cited 2020 Jun 23]. https://ddc.moph.go.th/viralpneumonia/file/guidelines/G_Invest_03_2.pdf

2. Thailand Ministry of Public Health, Department of Disease Control. Coronavirus disease 2019: COVID-19, updated on 23 March 2020 [cited 2020 Jun 23]. https://ddc.moph.go.th/viralpneumonia/file/g_srrt/g_srrt_250363.pdf

3. Thailand Ministry of Public Health, Department of Medical Sciences. Guideline: the laboratory diagnosis of COVID-19 [cited 2020 Aug 8]. https://ddc.moph.go.th/viralpneumonia/file/guidelines/G37.pdf

4. World Health Organization. Laboratory testing of 2019 novel coronavirus (2019-nCoV) in suspected human cases :interim guidance ,17 January2020]cited 2020Aug 8]. https://apps.who.int/iris/handle/10665/330676

5. Vandenbroucke JP, von Elm E, Altman DG, Gøtzsche PC, Mulrow CD, Pocock SJ, et al.; STROBE Initiative. Strengthening the Reporting of Observational Studies in Epidemiology (STROBE): explanation and elaboration. PLoS Med. 2007;4:e297. PubMed https://doi.org/10.1371/journal.pmed.0040297

6. Bursac Z, Gauss CH, Williams DK, Hosmer DW. Purposeful selection of variables in logistic regression. Source Code Biol Med. 2008;3:17. PubMed https://doi.org/10.1186/1751-0473-3-17

7. Azimi SS, Khalili D, Hadaegh F, Yavari P, Mehrabi Y, Azizi F. Calculating population attributable fraction for cardiovascular risk factors using different methods in a population based cohort study. J Res Health Sci. 2015;15:22–7. PubMed

8. Rückinger S, von Kries R, Toschke AM. An illustration of and programs estimating attributable fractions in large scale surveys considering multiple risk factors. BMC Med Res Methodol. 2009;9:7. PubMed https://doi.org/10.1186/1471-2288-9-7

9. Levine B. What does the population attributable fraction mean? Prev Chronic Dis. 2007;4:A14. PubMed

10. Mansournia MA, Altman DG. Population attributable fraction. BMJ. 2018;360:k757. PubMed https://doi.org/10.1136/bmj.k757

Appendix Table 1. Factors associated with severe acute respiratory syndrome coronavirus 2 infection in a multivariable model, including type of mask, among persons followed through contract tracing, March–April 2020, Thailand

Factors	Adjusted odds ratio (95% CI)†	p value
Sex		
F	Referent	0.35
M	0.75 (0.40–1.38)	
Age group, y		
≤15	0.56 (0.14–2.18)	0.19
>15–40	1.0 (referent)	
>40–65	1.77 (0.94–3.35)	
>65	1.00 (0.23–4.34)	
Contact place‡		
Nightclub	NA	–
Boxing stadium		
Workplace		
Household		
Others		
Shortest distance of contact		
Physical contact	Referent	0.02
≤1 m without physical contact	1.08 (0.57–2.03)	
>1 m	0.15 (0.04–0.66)	
Duration of contact within 1 m		
>60 min	Referent	0.10
>15–60 min	0.67 (0.29–1.56)	
≤15 min	0.25 (0.07–0.94)	
Sharing dishes or cups§		
N	Referent	0.39
Y	1.32 (0.69–2.53)	
Sharing cigarettes¶		
N	Referent	0.03
Y	3.48 (1.09–11.05)	
Handwashing#		
None	Referent	0.04
Sometimes	0.33 (0.14–0.79)	
Often	0.33 (0.12–0.87)	
Type of masks worn**		
None	Referent	0.54
Nonmedical masks only	1.29 (0.48–3.45)	
Nonmedical and medical	1.03 (0.26–4.07)	
Medical masks only	0.61 (0.25–1.49)	
Wearing masks all the time**		
N	Referent	0.02
Y	0.32 (0.12–0.82)	

*NA, not applicable; –, not available.
†Crude and adjusted odds ratios were estimated by using logistic regression with random effects for location and for index patient nested within the same location. Missing values were imputed using the imputation model.
‡The state enterprise office was included as a workplace. Others included restaurants, markets, malls, religious places, and households of index patients or other persons but not living together. Location was included in the model as a random effect variable.
§Sharing multiserving dishes and using communal serving utensils to portion food individually was not categorized as sharing dishes.
¶Included sharing electronic cigarettes and any vaping devices.
#Included washing with soap and water, and by using alcohol-based solutions.
**Wearing masks during the contact period. Wearing masks incorrectly, such as not covering both nose and mouth, was considered the same as not wearing a mask for analyses.

Appendix Table 2. Population attributable fraction of risk factors for severe acute respiratory syndrome coronavirus 2 infection in various locations based on contact tracing data, March–April 2020, Thailand*

	Nightclub		Boxing stadium		Workplace		Household		Other places		Overall	
Risk factors	Prev	PAF	Prev	PAF	Prev	PAF	Prev	PAF	Prev	PAF	Prev	PAF
Non-modifiable												
Female sex	0.51	0.08	0.13	0.002	0.40	0.03	0.68	0.09	0.40	0.08	0.45	0.03
Age group >15 y	1.00	0.32	0.98	0.05	0.99	0.37	0.82	0.26	0.96	0.37	0.95	0.15
Modifiable during the contact period												
Distance of contact <1 m	0.88	0.71	0.98	0.19	0.65	0.72	0.87	0.68	0.85	0.76	0.82	0.40
Duration of contact within 1 m >15 min	0.86	0.55	0.99	0.11	0.70	0.57	0.91	0.53	0.91	0.64	0.85	0.29
Sharing dishes or cups†	0.34	0.10	0.30	0.01	0.19	0.06	0.57	0.11	0.26	0.13	0.33	0.04
Sharing cigarettes‡	0.08	0.12	0.02	0.001	0.01	0.06	0	0	0.01	0.007	0.02	0.02
Not washing hands§	0.05	0.06	0.21	0.01	0.20	0.17	0.10	0.08	0.28	0.29	0.16	0.04
Not wearing masks all the time¶	0.60	0.52	0.80	0.08	0.78	0.65	0.86	0.55	0.94	0.68	0.78	0.28
Sum of all modifiable risk factors#		0.98		0.75		0.98		0.97		0.99		0.84

*Prevalence was estimated by using the imputed dataset. Population attributable fraction was estimated by using the direct method (*7*,*8*). PAF, population attributable fraction; Prev, prevalence.
†Sharing multiserving dishes and using communal serving utensils to portion food individually was not categorized as sharing dishes.
‡Included sharing electronic cigarettes and any vaping devices.
§Included washing with soap and water, and by using alcohol-based solutions.
¶Wearing masks incorrectly, such as not covering both nose and mouth, was considered the same as not wearing a mask for analyses.
#Age and gender were considered nonmodifiable risk factors; other risk factors were considered modifiable. Total PAF was directly estimated by using logistic regression in the form of natural logarithm; therefore, total PAF was not equal to the direct summation of PAF of each risk factor.

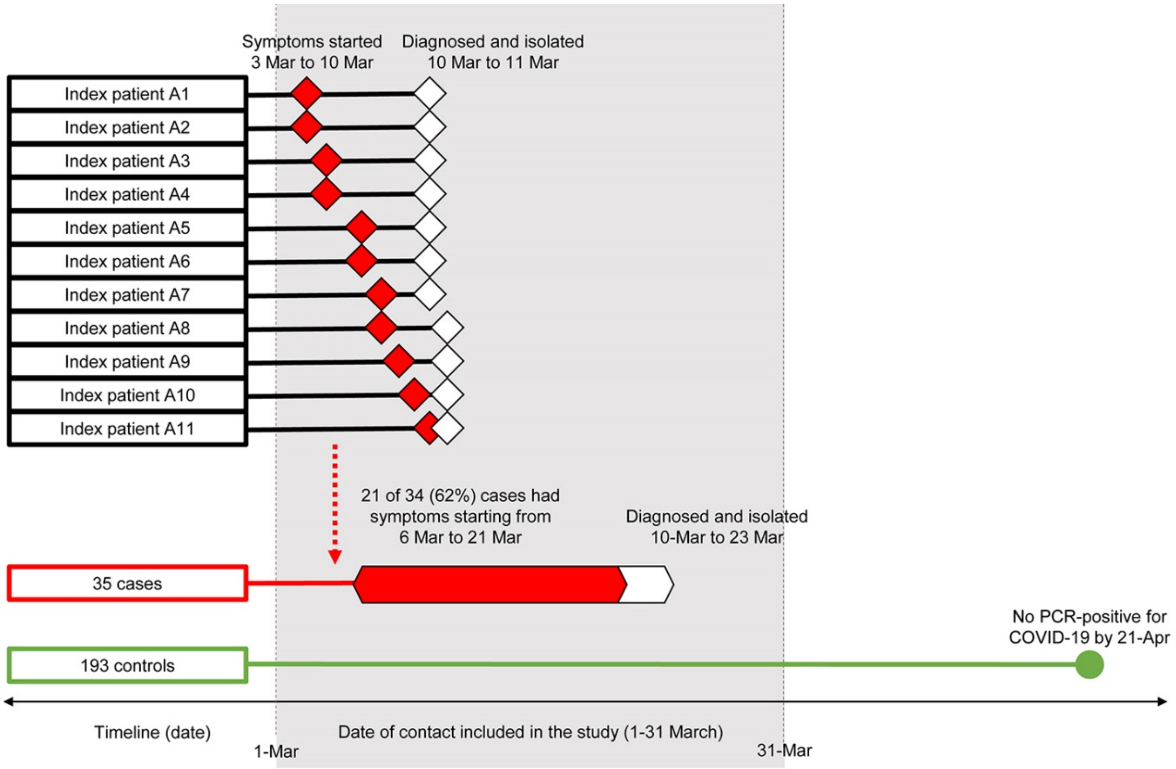

Appendix Figure 1. Timeline and possible transmission of severe acute respiratory syndrome coronavirus 2 from primary index patients of the nightclub cluster, March–April 2020, Thailand.

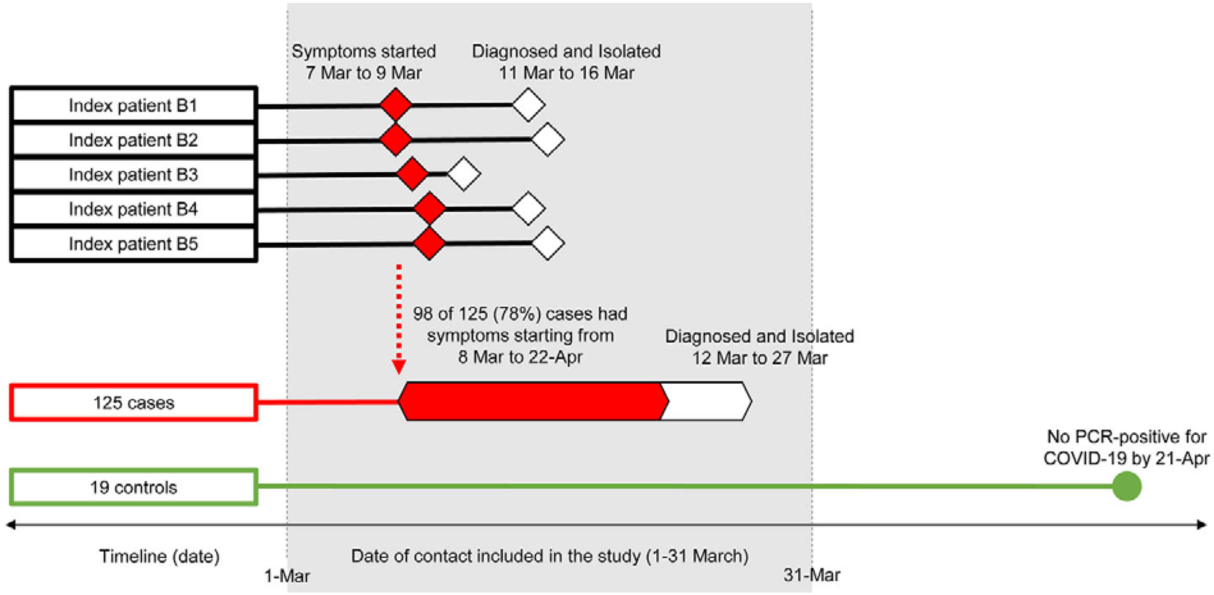

Appendix Figure 2. Timeline and possible transmission of severe acute respiratory syndrome coronavirus 2 from primary index patients of the boxing stadium state enterprise office cluster, March–April 2020, Thailand.

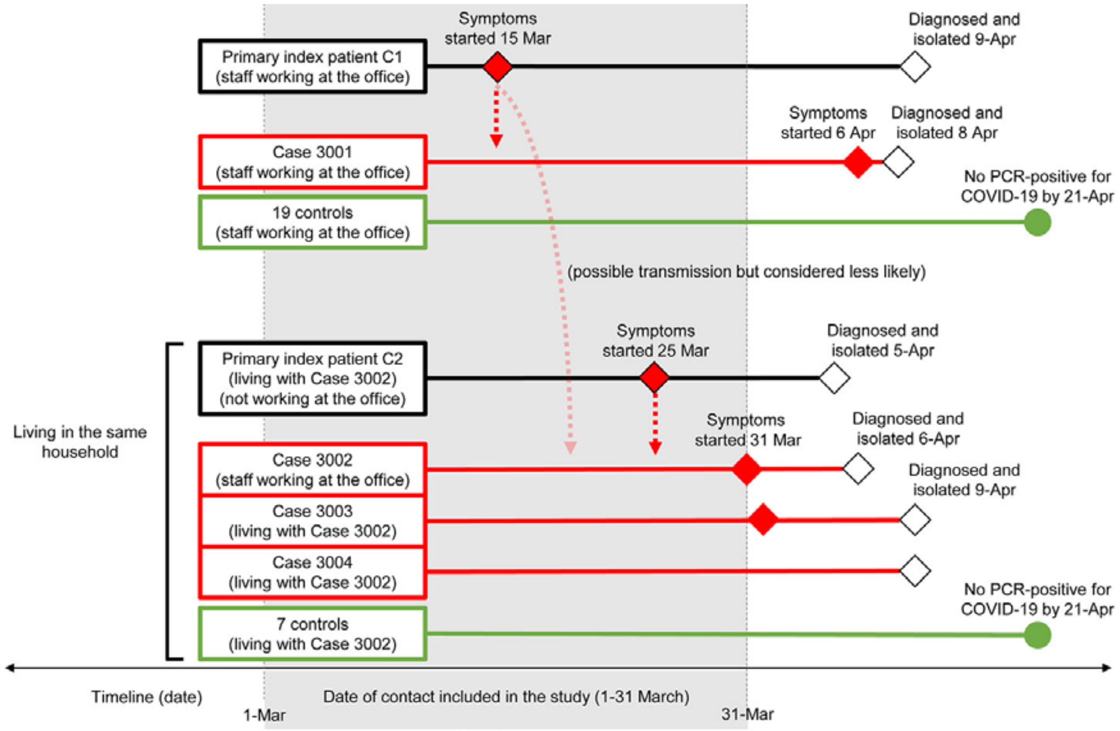

Appendix Figure 3. Timeline and possible transmission of severe acute respiratory syndrome coronavirus 2 from primary index patients of the state enterprise office cluster, March–April 2020, Thailand.
